# The lncRNAs involved in mouse airway allergic inflammation following induced pluripotent stem cell-mesenchymal stem cell treatment

**DOI:** 10.1186/s13287-016-0456-3

**Published:** 2017-01-06

**Authors:** Shu-Yue Wang, Xing-Liang Fan, Qiu-Ning Yu, Meng-Xia Deng, Yue-Qi Sun, Wen-Xiang Gao, Cheng-Lin Li, Jian-Bo Shi, Qing-Ling Fu

**Affiliations:** 1 Otorhinolaryngology Hospital, The First Affiliated Hospital, Sun Yat-sen University, Guangzhou, 510080 China; 2 Centre for Stem Cell Clinical Research and Application, The First Affiliated Hospital, Sun Yat-sen University, Guangzhou, 510080 China

**Keywords:** Asthma, Induced pluripotent stem cells-mesenchymal stem cells (iPSC-MSCs), Microarray, lncRNAs

## Abstract

**Background:**

We have previously reported that induced pluripotent stem cell (iPSC)-mesenchymal stem cells (MSCs) alleviated asthma inflammation in mice. Long noncoding RNAs (lncRNAs) were recently reported as being involved in the immune responses. However, whether lncRNAs are associated with iPSC-MSC immunomodulation in allergic inflammation is still unclear.

**Methods:**

Mice were induced into an asthmatic state and received treatment consisting of iPSC-MSCs. Memory T cells isolated from sensitized mice were challenged and co-cultured with iPSC-MSCs in vitro. Total RNA from the lungs and separated T cells were processed with an lncRNA/mRNA microarray. A series of bioinformatics technologies were used to screen the target lncRNAs.

**Results:**

iPSC-MSCs significantly prevented asthma inflammation and decreased the Th2 cytokine levels. Over 1300 lncRNAs were differentially expressed after the induction of asthma, and 846 or 4176 lncRNAs were differentially expressed with iPSC-MSC treatment in mice or in vitro, respectively. After overlapping the differentially expressed lncRNAs produced in a similar manner in mice and in vitro, 23 lncRNAs and 96 mRNAs were selected, in which 58 protein-coding genes were predicted to be potential targets of the 23 lncRNAs. Furthermore, using a series of bioinformatics technologies, 9 lncRNAs co-expressed with the most differentially expressed mRNAs, which were enriched in terms of the immune response, were screened out via Pearson’s correlation coefficient with mRNAs that were involved with inflammatory cytokines and receptors. lncRNAs *MM9LINCRNAEXON12105+* and *AK089315* were finally emphasized via quantitative real-time PCR validation.

**Conclusions:**

Our results suggested that aberrant lncRNA profiles were present after asthma induction and iPSC-MSC treatment, suggesting potential targets of allergic inflammation and iPSC-MSC-mediated immunomodulation.

**Electronic supplementary material:**

The online version of this article (doi:10.1186/s13287-016-0456-3) contains supplementary material, which is available to authorized users.

## Background

Asthma has become a global public health problem that negatively affects the quality of life of patients and causes a substantial medical and financial burden [1]. As an allergic airway inflammation disease, asthma is distinguished by a T lymphocyte imbalance and type 2 helper T (Th2) cell preponderance, airway hyper-reactivity, mucus hypersecretion, and reversible airway obstructions [2]. Allergy is also associated with dendritic cells (DCs), T regulatory (Treg) cells, and group 2 innate lymphoid cell (ILC2) dysfunction [3]. At present, a lot of reports are related to allergy pathogenesis [4]; however, reports about the noncoding RNA involved in allergic airway inflammation are limited.

Long noncoding RNAs (lncRNAs) are a group of functional RNA molecules with transcription lengths of more than 200 nucleotides which do not encode any protein products [5]. lncRNAs widely participate in physiological/pathological processes and human diseases, especially in tumorigenesis [6]. Hu et al. [7] reported that lncRNAs were differentially expressed in T cell development and differentiation. Moreover, lncRNAs were also reported as regulating the differentiation of DCs and the function of Treg cells [8, 9], which participate in CD4^+^ T-cell development and the activation process [10]. The expression profiles of lncRNAs were reported as being altered in eosinophilic esophagitis and in the CD8^+^ T cells of severe asthma patients [11, 12]. Therefore, we speculated that lncRNAs might be involved in airway allergic inflammation.

Mesenchymal stem cells (MSCs) have shown broad immunomodulatory properties [13, 14]. Bone marrow (BM)-derived MSCs (BM-MSCs) were reported to have immune regulation functions [15]. We have reported that MSCs derived from induced pluripotent stem cells (iPSCs), which have similar characteristics to BM-MSCs, effectively inhibited airway allergic inflammation in mice and significantly decreased immunoglobulin (Ig)E and Th2 cytokine expression levels [16]. Furthermore, our study found that iPSC-MSCs effectively improved peripheral blood T lymphocyte subsets in allergic rhinitis patients with immune imbalances [17]. However, the effects of iPSC-MSCs on lncRNAs in allergic airway inflammation remain to be further understood. Several studies reported on the lncRNA profiles in MSCs but not the targeted cells of MSCs [18]. The lncRNA expressions in the tissues or cells with the immunomodulation of MSCs were unclear. Here, we hypothesized that lncRNAs may be associated with iPSC-MSC immunomodulatory properties in airway allergic inflammation.

In this study, we induced a mouse model of asthma in which iPSC-MSCs were transplanted into the mice to study the lncRNAs involved in the allergy and MSC immunomodulatory process. Moreover, an in vitro model mimicking an allergy environment was also used. Both in vivo and in vitro studies identified differentially expressed lncRNAs that might be associated with the process of airway allergy and iPSC-MSC treatment. The identified lncRNAs were screened out using a series of bioinformatics technologies and validated by quantitative real-time polymerase chain reaction (qRT-PCR). Taken together, our results provide a key foundation for lncRNA functional studies in the fields of airway allergic inflammation and iPSC-MSC immunomodulatory properties. This is the first study that links lncRNAs with the effects of iPSC-MSCs in allergic airway inflammation.

## Methods

### Animals

Female BALB/c mice (4–6 weeks of age) were purchased from the Guangdong Medical Laboratory Animal Centre (Guangzhou, China). All procedures were performed according to the protocols approved by the Sun Yat-sen University Institutional Animal Care and Use Committee.

### Preparation and culture of pluripotent stem cell-mesenchymal stem cells

The human iPSC-MSCs used in this study were prepared in a similar way to our previous study, titled *Effects of mesenchymal stem cells from human iPS cells on differentiation, maturation, and function of dendritic cells,* which is under revision in *Stem Cell Research & Therapy*. Similar to general MSCs, iPSC-MSCs were positive for CD105, CD73, CD90, CD146, CD144, and CD44, and negative for CD34, CD14, and CD45. iPSC-MSC activities of osteogenic, chondrogenic, and adipogenic differentiation were confirmed. Detailed information on iPSC-MSCs is presented in Additional file 1.

### In vivo study

The mouse model of asthma was established according to our previous description with minor modifications [16, 19]. The mice were divided into three groups (*n* = 15, 16, and 15 for phosphate-buffered saline (PBS)/PBS/PBS, ovalbumin (OVA)/OVA/PBS, and OVA/OVA/iPSC-MSC, respectively). Airway responsiveness to methacholine (Mch), asthmatic inflammation in the lung, and RNA microarray extraction were analyzed. The details are presented in Additional file 1.

### In vitro study

The mice (*n* = 8) were sensitized by OVA on day 1 and day 7 and were sacrificed on day 14 to obtain the memory T (Tm) cells [20]. The Tm cells were stimulated with OVA and co-cultured with iPSC-MSCs. The interleukin (IL)-4 and IL-13 levels in the supernatant were measured. The details are presented in Additional file 1.

### lncRNA and mRNA microarrays

For the in vivo study (*n* = 3 for each group), total RNA for the lncRNA and mRNA microarrays was extracted from the lung tissues of three different groups (PBS/PBS/PBS, OVA/OVA/PBS, and OVA/OVA/iPSC-MSC). For the in vitro study (*n* = 3 for each group), total RNA for the lncRNA and mRNA microarrays was extracted from Tm cells that separated from the co-culture of two different groups (Tm + OVA and Tm + OVA + iPSC-MSC). The microarray service was provided by Arraystar (Arraystar, Rockville, MD, USA). The extract data were processed using the Agilent Feature Extraction Software.

### Cis and trans analyses

We predicted the potential targets of differentially expressed lncRNAs via cis and trans analyses. For the cis-acting analysis, we located the lncRNA positions with the RefSeq and UCSC Known Genes databases (mm10) within 10 kb of the known genes. For trans-acting analysis, we used BLAST software for the first round of screening (e < 1E-20) and the RNAplex software to choose trans-acting targets. The RNAplex parameters were set as –e-30.

### Pearsons’ correlation coefficient of lncRNA-mRNA

A Pearson’s correlation coefficient between the lncRNAs and mRNAs associated with the immune response was constructed. A Pearson’s correlation coefficient >0.6 or < –0.6 and *p* < 0.05 was considered correlative.

### Gene ontology and pathway analysis

A gene ontology (GO) analysis was performed to characterize genes and gene products in terms of the biological process (BP), cellular component (CC), and molecular function (MF). Fisher’s exact test was used to find if there was overlap between the differentially expressed list and the GO annotation list. The pathway analysis was a functional analysis that maps genes to KEGG pathways. This analysis was used to determine the main pathways in which differentially expressed mRNAs were enriched.

### RNA extraction and lncRNA quantification using qRT-PCR

Aberrant lncRNAs were finally selected from the lncRNA microarray results and were confirmed with additional samples using qRT-PCR (*n* = 6, 7, and 6 for PBS/PBS/PBS, OVA/OVA/PBS, and OVA/OVA/iPSC-MSC, respectively). The details are presented in Additional file 1. The primers used in the qRT-PCR are shown in Additional file 1: Table S1.

### Statistical analysis

The experimental data are expressed as the mean ± SEM. All statistical analyses were performed with SPSS software. For the Gaussian distribution data, one-way analysis of variance (ANOVA) followed by post-hoc Tukey (for equal homogeneity) or Dunnett T3 (for unequal homogeneity) tests were used for multiple comparisons between different groups. A Kruskal–Wallis rank sum test followed by a Mann–Whitney *U* test was performed for the comparisons that utilized abnormal distribution data. *p* < 0.05 was considered statistically significant.

## Results

### Identification of targeted lncRNAs

**Fig. 1:**
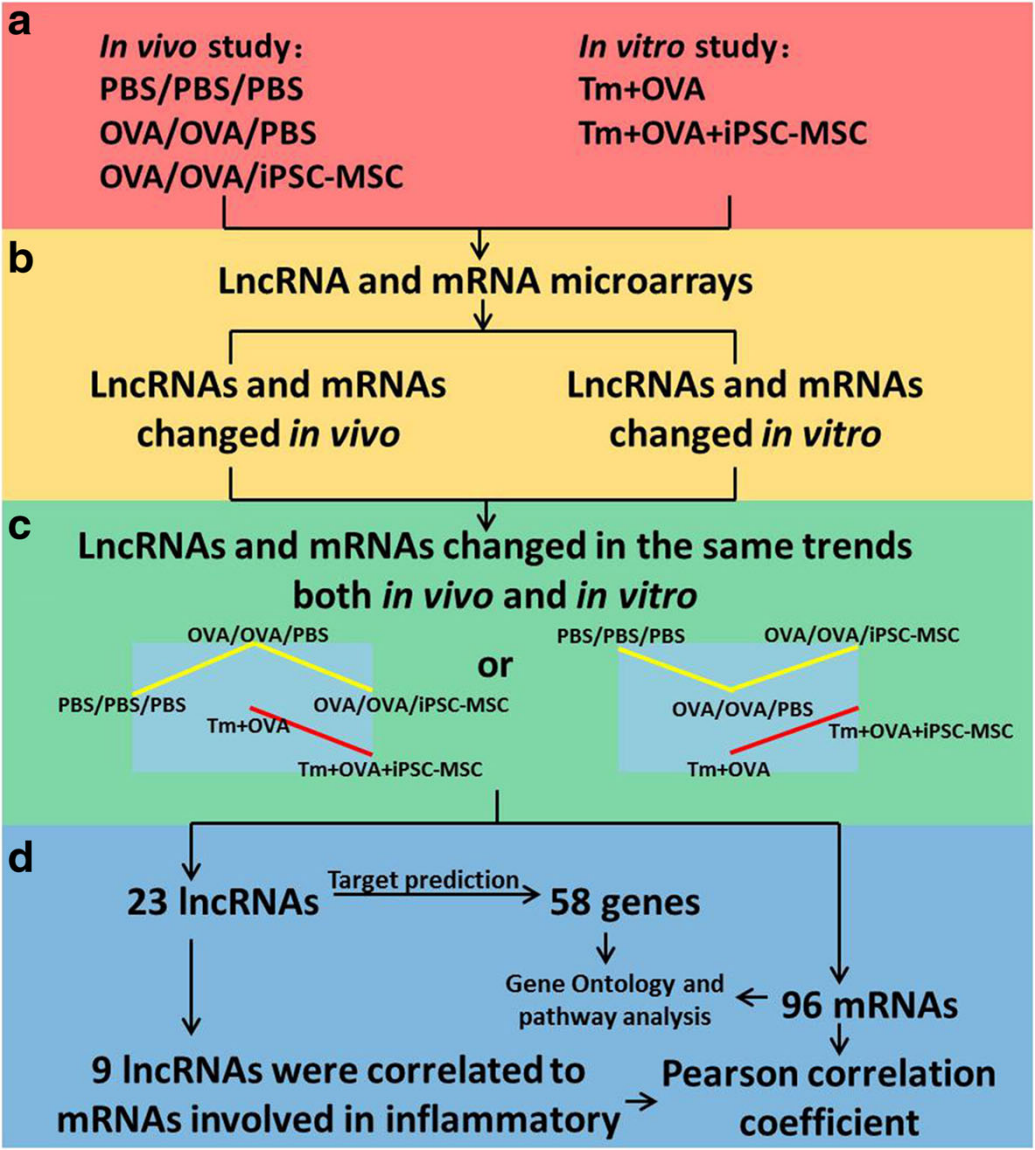
Identification of lncRNAs that were differently expressed as expected based on the lncRNA and mRNA microarray results. **a** Ovalbumin (*OVA*)-induced allergy was established both in vivo and in vitro. **b** Long noncoding RNAs (*lncRNAs*) and mRNAs that were differentially expressed in vivo and in vitro were isolated from the microarray data. **c** Twenty-three lncRNAs and 96 mRNAs expressed in the same manner (up or down after the induction of asthma and down or up with the treatment of induced pluripotent stem cell-mesenchymal stem cells (*iPSC-MSCs*)), both in vivo and in vitro, were selected. **d** Target prediction of the 23 lncRNAs was carried out; Pearson’s correlation coefficient with mRNAs involved in allergy was utilized to screen out 9 of the 23 lncRNAs. Gene ontology and pathway analysis of the co-expressed mRNAs was carried out to investigate the correlated functions of the genes. *PBS* phosphate-buffered saline, *Tm* memory T

Figure 1 summarized the systematic identification of lncRNAs in this study. We identified the possible lncRNAs that were involved in allergic inflammation and the immunomodulation of iPSC-MSCs. An OVA-induced allergy was established in both in vivo and in vitro environments, as described in the Materials and methods (Fig. 1a). lncRNAs and mRNAs that were differentially expressed in vivo and in vitro were isolated from the microarray data, respectively (Fig. 1b). Afterward, 23 lncRNAs and 96 mRNAs that were expressed in the same trend after the induction of asthma and with the treatment of iPSC-MSCs, both in vivo and in vitro, were selected (Fig. 1c). A series of bioinformatics technologies were used to further narrow down the allergy-specific candidate lncRNAs (Fig. 1d). Fifty-eight potential genes were predicted as targets of the selected 23 lncRNAs; nine lncRNAs that were correlated to the mRNAs involved in inflammation were further identified out of the 23. Pearson’s correlation coefficient between the 9 lncRNAs and 96 differentially expressed mRNAs was also calculated to confirm their co-expression relationship. To investigate the functions and pathways associated with 9 lncRNAs, we performed GO and a pathway analysis of both the 58 target genes and the 96 co-expressed mRNAs.

### iPSC-MSCs reduced airway inflammation in mice and decreased Th2 cytokine secretion in vitro

**Fig. 2:**
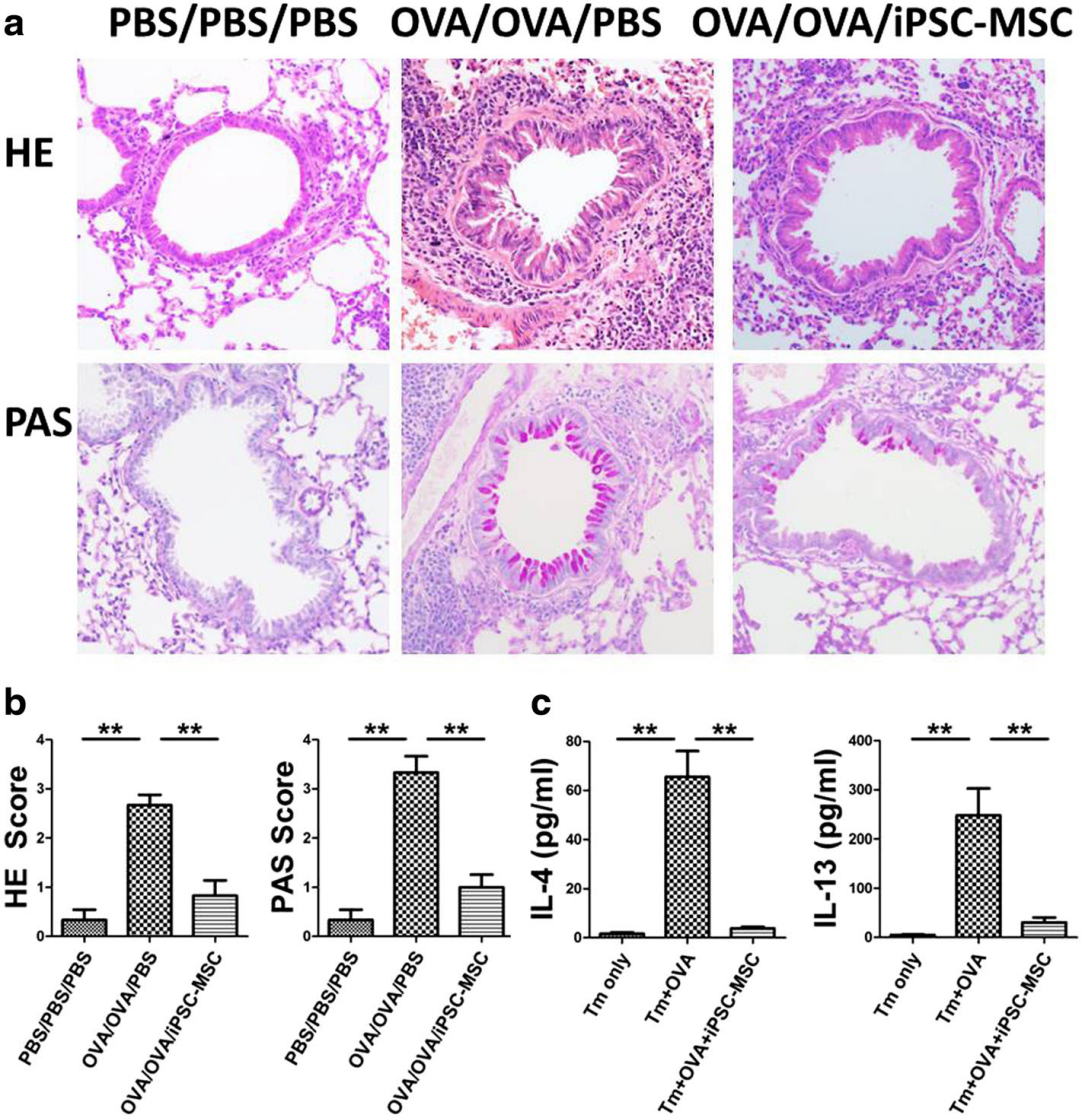
iPSC-MSCs alleviated airway allergy in vivo and reduced Th2 cytokines dramatically in vitro. **a** Representative photomicrographs of hematoxylin and eosin (*HE*)- and periodic acid-Schiff (*PAS*)-stained lung sections. Original magnification 200×. **b** The inflammatory infiltration and goblet cell hyperplasia was quantified by HE and PAS scores (*n* = 6). **c** Th2 cytokines IL-4 and IL-13 were detected in the supernatant. Memory T (*Tm*) cells were separated from the spleens of the mice and cultured in vitro (*n* = 5). **p < 0.01. *PBS* phosphate-buffered saline, *IL* interleukin, *iPSC-MSC* induced pluripotent stem cell-mesenchymal stem cell, *OVA* ovalbumin

Similar to our previous study [16, 19], the OVA/OVA/PBS group mice showed increased lung inflammatory infiltration compared to the PBS/PBS/PBS group (Fig. 2a). Moreover, the mouse models also showed higher airway hyperresponsiveness (AHR) levels at different Mch concentrations (6.25, 25, 50, and 100 mg/ml; Additional file 1: Figure S1). However, iPSC-MSC administration alleviated peribronchial inflammation (hematoxylin and eosin (H&E) staining) and decreased mucus secretion of hyperplastic goblet cells (periodic acid-Schiff (PAS) staining) (Fig. 2a), and significantly inhibited AHR (Additional file 1: Figure S1). Pathological scoring (H&E and PAS) in the OVA/OVA/iPSC-MSC group was decreased two- to threefold compared to the OVA/OVA/PBS group (Fig. 2b). We also observed that iPSC-MSCs significantly decreased the serum IgE level and Th2 cytokine levels (IL-4, IL-5, and IL-13) in the lavage fluid (data not shown). These results confirmed our previous study that iPSC-MSC treatment was effective in murine airway allergic inflammation [16].

To further identify the effects of iPSC-MSCs on Th2 responses and to identify the possible lncRNAs involved in the immunomodulation of iPSC-MSCs from the huge amount of microarray data, we used an in vitro model to mimic the Th2 environment. The Tm cells were isolated and purified from the spleen mononuclear cells of mice, which were sensitized twice using OVA and then were further stimulated with OVA in culture systems. We found that both IL-4 and IL-13 (Fig. 2c), but not IL-5 with undetectable levels (data not shown), were significantly upregulated after being stimulated by OVA compared to the Tm only group (both *p* < 0.01; Fig. 2c). However, the administration of iPSC-MSCs dramatically reduced these cytokine levels (both *p* < 0.01; Fig. 2c), similar to our in vivo results.

### mRNA and lncRNA expression profiles in allergy and iPSC-MSC immunomodulation

**Fig. 3:**
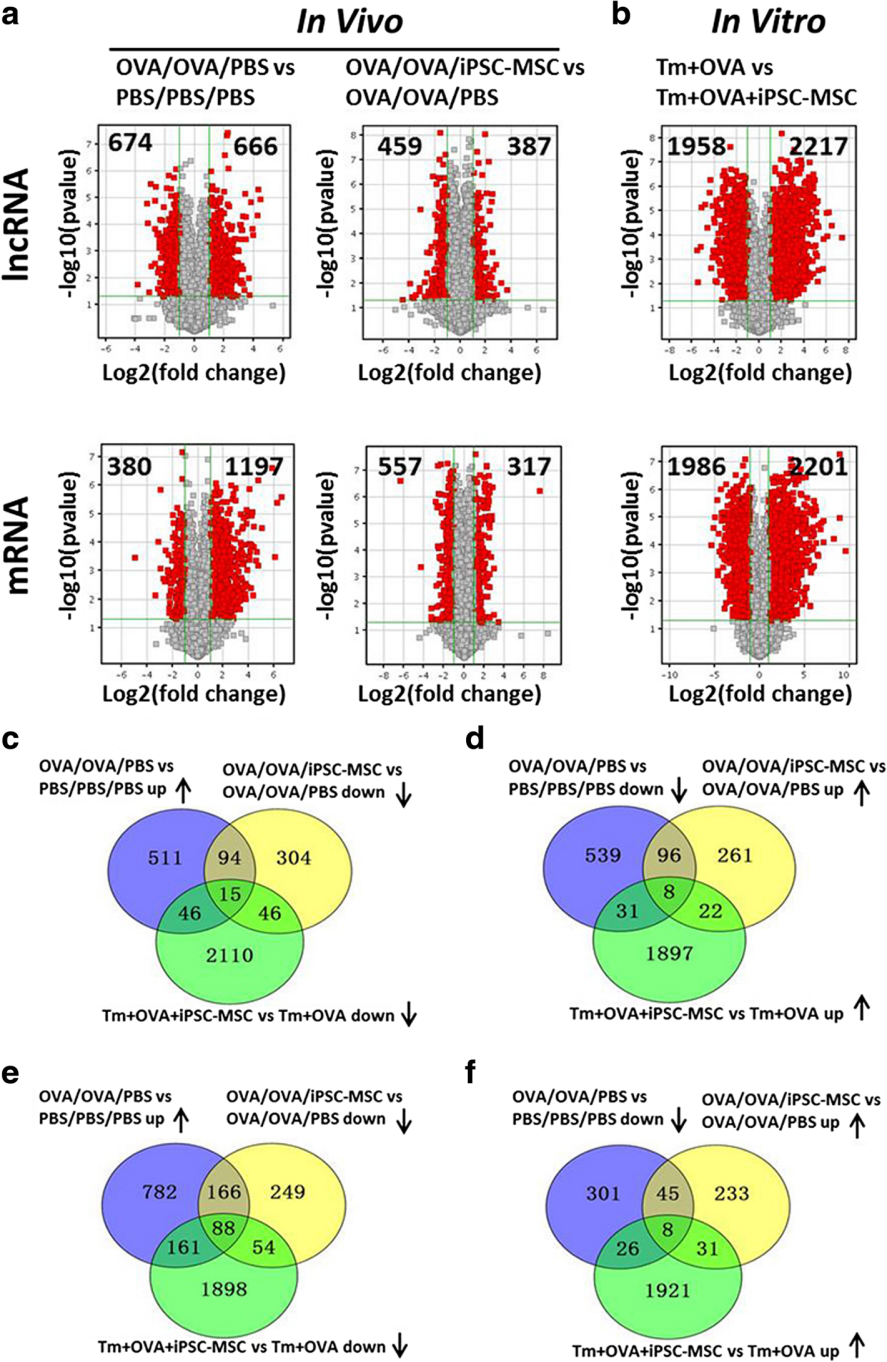
lncRNAs and mRNAs that were expressed in the same manner in vivo and in vitro were selected. **a** A Volcano plot provided the fold-change and *p* values of differentially expressed long noncoding RNAs (*lncRNAs*; *top*) and mRNAs (*bottom*). The vertical lines represent a twofold change in expression (up or down), and the horizontal lines represent *p* values = 0.05. Pairwise comparisons between the OVA/OVA/PBS group and PBS/PBS/PBS group (*left*) and between the OVA/OVA/PBS group and OVA/OVA/iPSC-MSC group (*right*) are shown. The *red* points represent differentially expressed lncRNAs or mRNAs with statistical significance (*n* = 3). **b** A Volcano plot provided fold-change and *p* values of differentially expressed lncRNAs (*top*) and mRNAs (*bottom*) between the memory T (*Tm*) + OVA group and Tm + OVA + iPSC-MSC group (*n* = 3). **c**–**f** Venn diagrams provided a detailed distribution of differentially expressed lncRNAs (**c**, **d**) and mRNAs (**e**, **f**) in two patterns (up then down in vivo and down in vitro (**c**, **e**), down then up in vivo and up in vitro (**d**, **f**)). The three pies represent differentially expressed lncRNAs and mRNAs of different combinations between the OVA/OVA/PBS group and PBS/PBS/PBS group (*blue*), between the OVA/OVA/iPSC-MSC group and OVA/OVA/PBS group (*yellow*), and between the Tm + OVA group and Tm + OVA + iPSC-MSC group (*green*). The overlap of the three pies was selected. *PBS* phosphate-buffered saline, *iPSC-MSC* induced pluripotent stem cell-mesenchymal stem cell, *OVA* ovalbumin

mRNA and lncRNA expression profiles were measured via microarrays. The lncRNA microarray covered more than 24,000 lncRNAs in mice. As shown in Fig. 3, 1340 lncRNAs were observed as differentially expressed after the induction of airway allergic inflammation, with 666 lncRNAs upregulated and 674 lncRNAs downregulated in the OVA/OVA/PBS group compared to the PBS/PBS/PBS group. A total of 846 lncRNAs were observed to be differentially expressed after the treatment of iPSC-MSCs, with 387 lncRNAs upregulated and 459 lncRNAs downregulated in the OVA/OVA/iPSC-MSC group compared to the OVA/OVA/PBS group. A total of 4176 lncRNAs were observed to be differentially expressed between the Tm + OVA group and the Tm + OVA + iPSC-MSC group, with 2217 lncRNAs upregulated and 1958 lncRNAs downregulated (Fig. 3a; *p* < 0.05 and fold change >2).

The key lncRNA regulators that presented the reverse variation trends between asthma induction and iPSC-MSC transplantation should have more significance for our exploration of the possible mechanisms of MSC-mediated immunomodulation. Therefore, we next selected two patterns with opposite directions (up then down or down then up) after the asthma induction and after iPSC-MSC treatment for further study (Fig. 3c, d). However, there were still 109 aberrant lncRNAs for the pattern of up then down (Fig. 3c) and 104 aberrant lncRNAs for the pattern of down then up (Fig. 3d). Therefore, to further narrow the scope of the selected lncRNAs, we created an overlap for the similar patterns of the differentially expressed lncRNAs in mice and in vitro. One pattern was the overlap of the lncRNAs that increased after the induction of allergic airway inflammation but decreased with the treatment of iPSC-MSCs in mice (up then down) and the lncRNAs that decreased with the treatment of iPSC-MSCs in Tm cells in vitro (down) (Fig. 3c). Another pattern was the overlap of the aberrant lncRNAs with down then up in mice and the aberrant lncRNAs that were up in vitro (Fig. 3d). Finally, a total of 23 lncRNAs involved in the immunomodulation of iPSC-MSCs in allergy were selected. Specifically, 15 lncRNAs had the pattern of up then down in mice and down in vitro (Fig. 3c), and 8 lncRNAs had the pattern of down then up in mice and up in vitro (Fig. 3d). Detailed information, including seqname, source database, chromosome localization, etc, regarding the 23 selected and differentially expressed lncRNAs is shown in Additional file 1: Table S2. The variation trends for the above 23 lncRNAs with two different patterns are further clearly shown in Additional file 1: Figure S2.

Similarly, we found that a total of 88 mRNAs were differentially expressed with a pattern of up then down in mice and down in vitro (Fig. 3e). Eight mRNAs were differentially expressed with a pattern of down then up in mice and up in vitro (Fig. 3f).

**Fig. 4:**
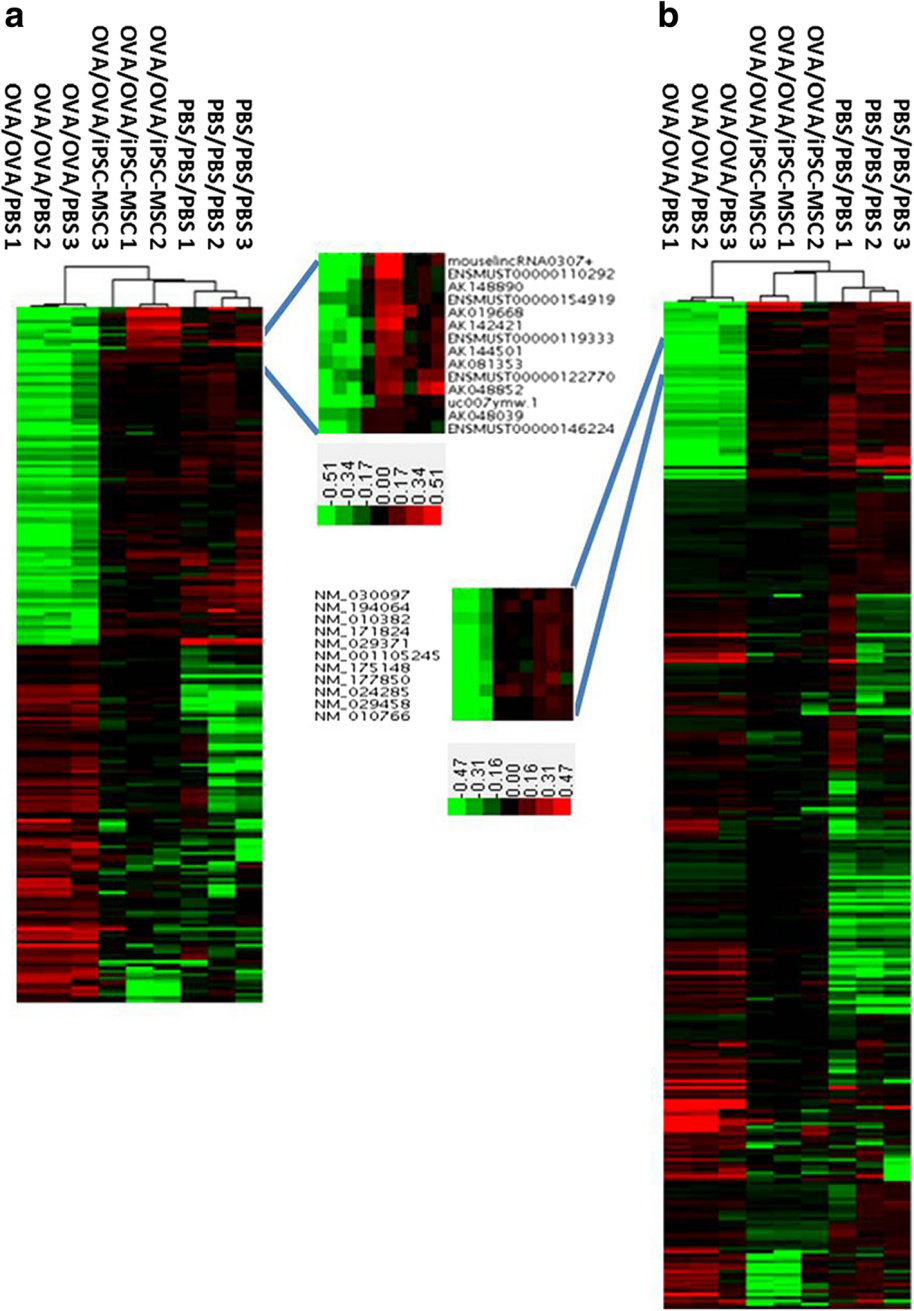
The differentially expressed lncRNAs associated with allergy and iPSC-MSC treatment. Hierarchical cluster analysis of aberrant lncRNAs (**a**) and mRNAs (**b**) whose expressions were changed in OVA/OVA/PBS and restored in OVA/OVA/iPSC-MSC (*n* = 3 for each group). *Red* indicates high relative expression and *green* indicates low relative expression. *PBS* phosphate-buffered saline, *iPSC-MSC* induced pluripotent stem cell-mesenchymal stem cell, *OVA* ovalbumin

The pathogenesis of allergic inflammation is a complex progress associated with various immunocyte dysfunctions, including T lymphocytes, ILCs, DCs, etc. To confirm the expression patterns of lncRNAs and mRNAs associated with allergy and iPSC-MSC immunomodulation comprehensively, hierarchical clustering was performed to analyze the expressions of 213 lncRNAs and 307 mRNAs in vivo. The data showed a distinguishable lncRNA and mRNA expression profiling pattern between the OVA/OVA/PBS group and the OVA/OVA/iPSC-MSC group (Fig. 4). The heat map provided a viable solution for showing the expression patterns of aberrant lncRNAs and mRNAs; moreover the hierarchical clustering results were also helpful for analyzing the interactions and relationships of different RNAs for the subsequent research.

### Cis and trans analysis

To predict the potential targets of differentially expressed lncRNAs involved in allergy and iPSC-MSC immunomodulation, cis and trans analyses of the selected 23 lncRNAs were performed. A cis-acting analysis was used to predict the targets of lncRNAs; a major function of lncRNAs is regulating the neighboring mRNA expression through cis-acting mechanisms. For the cis-acting analysis, the genomic position of the selected 23 lncRNAs relative to the genomic positions of their closest known genes were determined from the RefSeq and UCSC Known Genes databases [21]. We searched the genes in the chromosome 10 kb upstream and downstream of 23 lncRNAs, in which 14 potential targets were identified. For a trans-acting analysis, we determined the potential target genes of differentially expressed lncRNAs with a program known as RNAplex, which reduced the time needed to localize putative hybridization sites, mainly by neglecting intramolecular interactions and using a lightly simplified energy model [22]. The trans role of 23 selected lncRNAs was examined with BLAST software and the RNAplex software to select trans-acting targets, and 44 protein-coding genes were identified. In total, 58 genes were predicted as targets of the 23 selected lncRNAs (Additional file 1: Figure S3A). The results of the target prediction provided a foundation to research the interaction of lncRNAs and mRNAs, and some potential target genes that were predicted may be the key regulators of lncRNA-mediated allergy regulation and iPSC-MSC immunomodulation. Therefore, we conducted a GO and pathway analysis of 58 predicted genes, and all significant GO categories and pathways are shown in Additional file 1: Figure S3B. Most of the predicted genes were enriched in GO categories, including ras protein signal transduction, cation binding, and the ncRNA metabolic process, etc. A KEGG pathway analysis indicated that the genes were involved in the transforming growth factor (TGF)-beta signaling pathway and aminoacyl-tRNA biosynthesis (Additional file 1: Figure S3B).

### Co-expression and correlation of lncRNA and mRNA

**Fig. 5:**
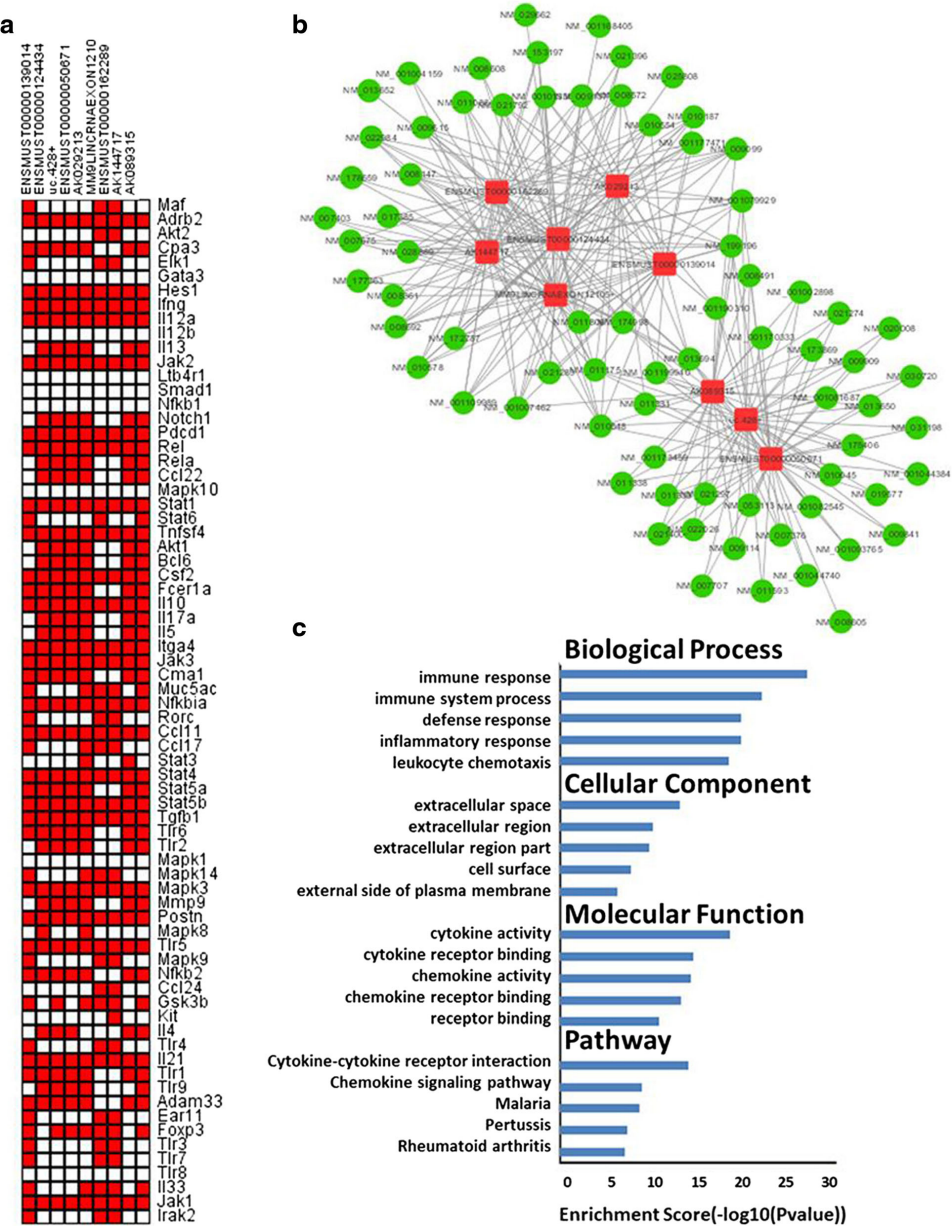
The nine lncRNAs were co-expressed with mRNAs, which were enriched in the immune response. **a** Pearson’s correlation coefficient of 72 mRNAs (*vertical axis*) involved in inflammatory cytokines and receptors, together with 23 screened-out lncRNAs was taken. Nine lncRNAs (*horizontal axis*) were isolated, in which >40 co-expressed mRNAs were selected. Pearson’s correlation coefficients >0.6 or < –0.6, and *p* values <0.05 were considered correlative. *Red* represents a co-expressed pair, and *white* does not. **b** The co-expression network was established between the selected nine lncRNAs and 96 mRNAs. The *red* nodes denote lncRNAs, and the *green* nodes denote mRNAs. Pearson’s correlation coefficients >0.6 or < –0.6, and *p* values <0.05 were considered correlative. **c** GO and pathway analyses of 96 mRNAs co-expressed with lncRNAs were performed. The ontology covers three domains: biological process, cellular component, and molecular function. The top five significant GO categories and pathways are shown

To further identify the lncRNAs that were closely connected to an immune response, a correlation analysis of 23 selected lncRNAs and mRNAs involved in allergic inflammation and iPSC-MSC immunomodulation was carried out. Seventy-two mRNAs, including *Il4*, *Il5*, *Il13*, *Il10*, *Jak1*, *Jak3*, *Stat6*, *Gata3*, *Foxp3*, *Ccl17*, and *Ccl22* which related to inflammatory cytokines and receptors, were chosen to confirm their relationships with the 23 selected lncRNAs. Each of the selected lncRNAs was used to calculate the Pearson’s correlation coefficient and *p* value with 72 mRNAs; nine out of 23 lncRNAs that had more than 40 co-expressed mRNAs were screened out (Fig. 5a; Pearson’s correlation coefficient >0.6 or < –0.6, *p* < 0.05).

The 96 differentially expressed mRNAs in iPSC-MSC immunomodulation were chosen to confirm their relationships with the nine screened-out lncRNAs. The Pearson’s correlation coefficient between the nine lncRNAs and 96 mRNAs was also calculated (Pearson’s correlation coefficient >0.6 or < –0.6, *p* value <0.05). These lncRNAs had a co-expression with most of the 96 mRNAs; a pattern of high numbers of connections between protein-coding mRNAs and lncRNAs is also observed in a visual solution (Fig. 5b). We found that these nine lncRNAs were co-expressed with 74 protein-coding mRNAs, including *Il4*, *Il10*, *Il1*α, *Il1*β, *Ccl2*, *Ccl4*, *Ccl9*, *Ccl22*, and *CD14*, which were associated with the immune response. Moreover, these nine lncRNAs may participate in airway allergic inflammation and iPSC-MSC immunomodulation through these mRNAs.

### Gene ontology and pathway analysis of mRNA expression

To investigate whether the clustering of genes correlated with specific functions and pathways, we performed a GO and pathway analysis of the differentially expressed mRNAs which were co-expressed with the nine screened-out lncRNAs. The GO and KEGG analyses of these mRNAs revealed that major enriched pathways were associated with immune responses, cytokine activity, cytokine-cytokine receptor interactions, and chemokine signaling pathways. The ontologies covered three domains: BP, CC, and MF; the top five significant GO categories and pathways are shown in Fig. 5c. We found that the most enriched BP ontologies targeted by lncRNAs that co-expressed mRNAs were the immune response, immune system process, defense response, inflammatory response, and leukocyte chemotaxis. The most enriched CC ontologies were the extracellular space, extracellular region, extracellular region part, cell surface, and the external side of the plasma membrane. The most enriched MF ontologies were cytokine activity, cytokine receptor binding, chemokine activity, chemokine receptor binding, and receptor binding. The KEGG pathway analysis indicated that the lncRNA co-expressed mRNAs were involved in cytokine-cytokine receptor interactions, chemokine signaling pathways, malaria, pertussis, and rheumatoid arthritis. The results of the GO and KEGG pathway analysis suggested that lncRNAs may be associated with an immune response.

### Quantitative real-time-PCR

**Fig. 6:**
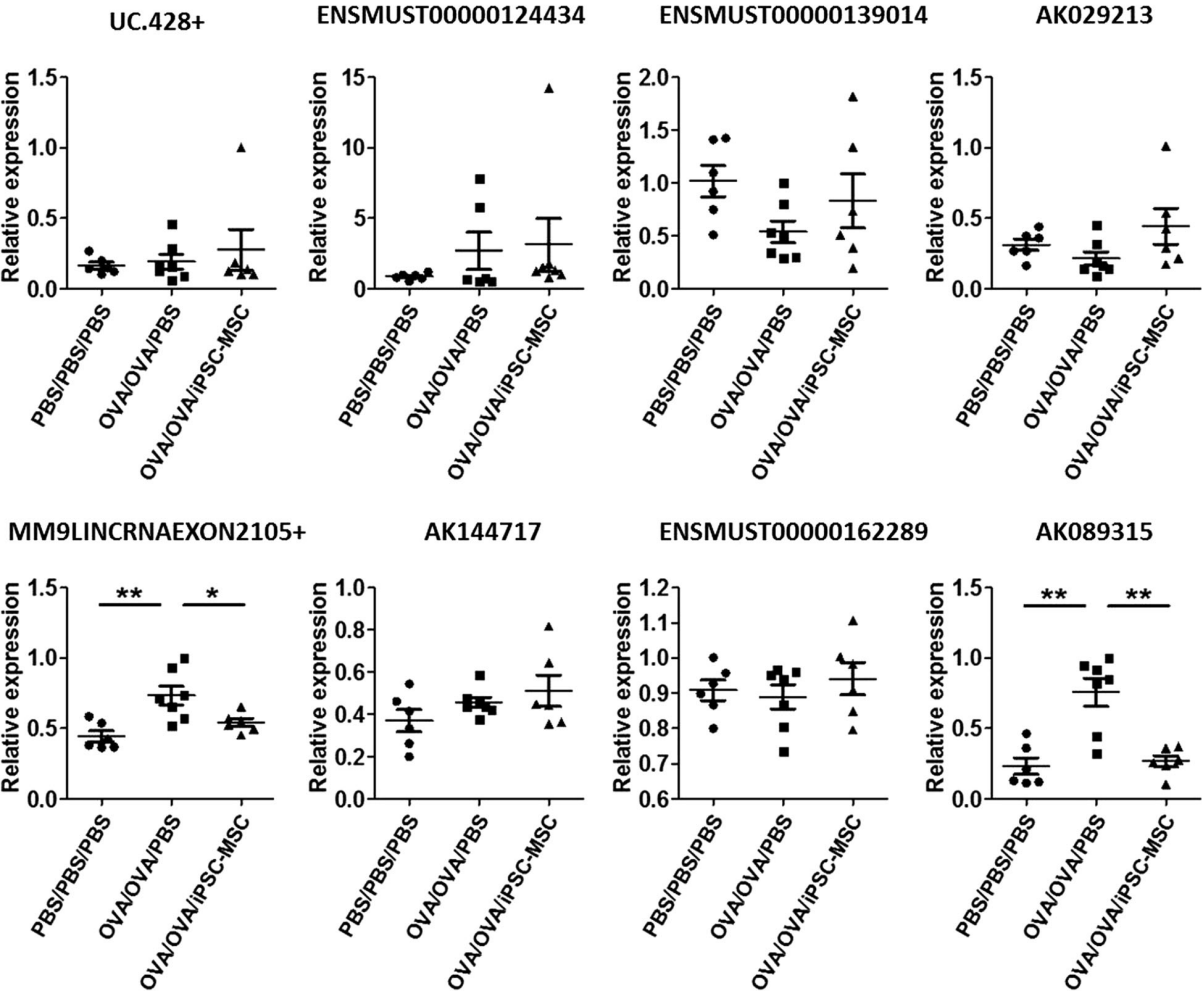
Eight selected lncRNA expression levels were validated in vivo. The expressions of the selected eight lncRNAs were validated by qRT-PCR in three groups in vivo. Two lncRNAs, *MM9LINCRNAEXON12105+* and *AK089315*, were upregulated in the allergy reaction and downregulated in iPSC-MSC immunomodulation in vivo. *n* = 6, 7, and 6; *p < 0.05, **p < 0.01. *PBS* phosphate-buffered saline, *iPSC-MSC* induced pluripotent stem cell-mesenchymal stem cell, *OVA* ovalbumin

To confirm the highest correlative lncRNAs with airway allergic inflammation and iPSC-MSC immunomodulation, qRT-PCR was used to validate the nine screened-out lncRNAs by utilizing lung samples from the PBS/PBS/PBS, OVA/OVA/PBS, and OVA/OVA/iPSC-MSC groups (Fig. 6). Two lncRNAs, *MM9LINCRNAEXON12105+* and *AK089315*, were identified because their expressions were the best in terms of conformance and stability, similar to those obtained from the microarray analysis. The above two lncRNAs showed significant differences in three groups in vivo, which were upregulated in asthma and downregulated in the iPSC-MSC treatment. Furthermore, two other lncRNAs, *ENSMUST00000124434* and *AK029213*, showed trends as expected, which were downregulated after asthma induction and upregulated after iPSC-MSC treatment, but the results were not statistically significant, probably because of the limited sample size. The results indicated that *MM9LINCRNAEXON12105+* and *AK089315* may be the potential regulators of allergy and the targets of iPSC-MSC immunomodulation (the primer specificity of one lncRNA,*mouselincRNA0307+*, was not high enough, so only eight are shown in Fig. 6).

## Discussion

Adult MSCs, including BM-MSCs, exhibit several inherent defects that hinder their regenerative efficiency for standard clinical applications, such as a limited proliferative capacity and rapid loss of differentiation potential [23]. Moreover, MSCs present high batch-to-batch variability due to differences in donor age, health conditions, and genetic background [24]. iPSC-MSCs not only show much greater proliferation and differentiation capacity but also have low immunogenicity compared to BM-MSCs [25], which overcomes the disadvantages of adult MSCs. Therefore, iPSC-MSCs that have broader prospects in medical application were used in our research.

In this study, the expression profiles of mRNAs and lncRNAs in asthma induction and iPSC-MSC treatment were examined via microarray, in which 23 lncRNAs and 96 mRNAs were identified as being differentially expressed in the same manner between in vivo and in vitro. Fifty-eight protein-coding genes were predicted as potential targets of the 23 lncRNAs. Nine out of 23 lncRNAs were selected via a correlation analysis, with 72 protein-coding genes involved in inflammatory cytokines and receptors. A correlation analysis confirmed the relationships between the nine selected lncRNAs and the 96 differentially expressed mRNAs, most of which were enriched in the immune process. Finally, lncRNAs *MM9LINCRNAEXON12105+* and *AK089315* were emphasized via qRT-PCR validation. These lncRNAs may be the potential targets of allergic reactions and MSC-mediated immunomodulation.

lncRNAs have been proven to play crucial roles in diverse processes and diseases via regulating protein-coding gene expressions [8]. For instance, the famous lncRNA *MALAT1* was proven to indicate a hint of tumor metastasis [26]. In recent years, some evidence has been found to show that lncRNAs are important regulators of the immune response [27]. They were not only involved in the innate immune response but also participated in T-cell regulation and host-pathogen interactions [28–30]. Xia et al. [10] found that the expression profiles of lncRNAs in different stages of CD4^+^ T cells are distinguishable. It was demonstrated that changes in lncRNA expression were associated with severe asthma in circulating CD8^+^ T cells [11]. The lncRNA BANCR was upregulated in eosinophilic esophagitis, which is another allergic inflammatory disorder, and was induced in IL-13 that treated primary esophageal epithelial cells [12]. However, lncRNA profiles in asthma which are dominant in terms of Th2 responses, remain unclear. Using the lncRNA microarray, we identified that 1340 lncRNAs were differentially expressed after the induction of asthma, with 666 lncRNAs being upregulated and 674 lncRNAs downregulated. Using a series of bioinformatics technologies, we further studied the possible nine lncRNAs co-expressed with the most differentially expressed mRNAs enriched in the immune response. We finally confirmed using qRT-PCR that the lncRNAs *MM9LINCRNAEXON12105+* and *AK089315* increased after the induction of asthma. We acknowledge that there should be more lncRNAs involved in the allergic airway inflammation aside from the lncRNAs selected for the overlap with the treatment of iPSC-MSCs in our study. Our study provides clear evidence that there are different lncRNA expressions for asthma, especially for T cells, suggesting their potential role in the immunopathogenesis and therapy of these diseases.

We previously reported that iPSC-MSCs significantly prevent allergy airway inflammation and regulate the T-lymphocyte responses in allergic rhinitis [17]. Some studies reported that lncRNAs played a role in the maintenance and differentiation of MSCs for mature lineages [31], and one report described lncRNA expression patterns in TLR3 that was stimulated in adipose tissue-derived MSCs [18]. However, previous studies focused on the lncRNA profiles in MSCs but not on the targets of MSCs. There are no reports on the lncRNA expression profiles after the treatment of MSCs, even for BM-MSCs on immune cells or in immune/allergic diseases. In this study, we identified that 846 or 4176 lncRNAs were differentially expressed after the iPSC-MSC treatment in mice or in the in vitro model, respectively. After a series of analyses, nine lncRNAs co-expressed with the most differentially expressed mRNAs enriched in the immune response were screened out, and the lncRNAs *MM9LINCRNAEXON12105+* and *AK089315* were finally confirmed by qRT-PCR to increase with the iPSC-MSC treatment. We believe that our study is the first report that links lncRNAs with MSCs, especially iPSC-MSC immunomodulatory properties.

Our microarray analysis identified that a total of 213 lncRNAs were differentially expressed in opposite directions (down then up or up then down) after the asthma induction and after iPSC-MSC treatment. However, the number of candidate lncRNAs was still too large. An in vitro study was supplemented to narrow the scope. IL-4 and IL-13, but not IL-5, were highly secreted by the sensitized Tm cells after challenges in the culture. These results prove that we utilized a proper in vitro model for allergy research. Moreover, for the first time ever, we demonstrated that iPSC-MSCs dramatically decreased the levels of IL-4 and IL-13 in *an* ex vivo condition. To identify lncRNAs associated with the allergy reaction and iPSC-MSC immunomodulation, two types of microarray patterns were selected. One was upregulated after the induction of allergic airway inflammation, downregulated with the treatment of iPSC-MSCs in mice, and downregulated with the treatment of iPSC-MSCs in Tm cells in vitro (up then down in mice and down in vitro). Another was completely the opposite (down then up in mice and up in vitro). Twenty-three lncRNAs were screened out via the overlap of the in vivo study and in vitro study. Furthermore, nine out of 23 lncRNAs were identified as being involved in the immune response after the analysis of the target prediction, GO, and pathway analysis, and Pearson’s correlation coefficient.

We also carried out an mRNA microarray at the same time. mRNAs involved in allergy and iPSC-MSC treatment were also identified, and their relationships with the lncRNAs were analyzed. Among the 72 mRNAs co-expressed with the selected 23 lncRNAs, some were closely associated with allergic inflammation, such as: Th2 cytokine coding genes, including *Il4*, *Il5*, and *Il13* [32]; Th2-specific transcription factors, including *Stat5* and *Stat6* [33]; Treg transcription factors, including *Foxp3* [34]; and chemokine coding genes such as *Ccl17* and *Ccl22*, which play a critical role in the recruitment and activation of Th2 cells [35]. The protein-coding genes listed were closely linked to allergy and associated with lncRNAs; moreover, some of them such as *Il4*, *Il5*, and *Il13* were regulated by iPSC-MSC treatment [16], and therefore these lncRNAs were also associated with iPSC-MSC immunomodulation. Our data confirm that lncRNAs are involved in asthma inflammation in mice, and they are also involved in the immunomodulation process when treated by iPSC-MSCs.

Despite accumulating evidence suggesting that the majority of lncRNAs are likely to be functional [36, 37], we also identified more than 200 lncRNAs that were involved in iPSC-MSC immunomodulation. However, only nine lncRNAs have been shown to be relevant. Finally, by using additional lung samples, two lncRNAs, *MM9LINCRNAEXON12105+* and *AK089315*, were proven to be the most relevant because they differentially expressed significant differences in the airway allergy and iPSC-MSC treatment. For further functional study, genomic modifications of the above two selected lncRNAs could be used to explore their functions in identified mRNA regulation. The targeted lncRNA knockout or overexpressed T cells that were challenged and co-cultured with iPSC-MSCs in vitro could be processed with a microarray or via RNA-seq analysis to verify that the mRNAs are regulated by a specific lncRNA. The results matched the identified mRNAs associated with allergy, and iPSC-MSC treatment should be selected. The mRNAs mediated by two selected lncRNAs would be determined, and the lncRNA/mRNA pathway involved in the allergic reaction and iPSC-MSC immunoregulation could be verified. We have attempted to perform genomic modification of the two target lncRNAs in murine primary Tm cells with small-interfering RNA. However, primary T lymphocytes are generally resistant to non-viral vector-based transfection methods [38]. Significant time and effort is still needed to solve this problem. This part of the work will need to be completed in the future.

In our study, the in vitro study was supplemented to help narrow the scope of lncRNAs selected from the in vivo data. The advantage of this was effective in focusing on the target lncRNAs. However, the limitation was that the samples between the in vivo and in vitro study were different. The lungs used for the in vivo study not only contained T lymphocytes, which were used for the in vitro study, but also included other types of cells such as epithelial cells, ILC2, and DCs. Therefore, the selected lncRNAs in this study still focused on T lymphocytes. The selected lncRNAs involved in the allergy and iPSC-MSC immunomodulation may also be associated with other types of cells, such as epithelial cells and DCs. Moreover, MSCs were also shown to regulate epithelial cells, DCs, etc. [39, 40], which is a research emphasis for the future.

## Conclusions

In conclusion, our study found that airway allergic inflammation can cause alterations of lncRNAs expressed in mice, and some of these altered lncRNAs are also involved in the alleviation of iPSC-MSC airway inflammation. This study evaluated the aberrant lncRNA expression profiles both in asthma and iPSC-MSC treatment, which demonstrated that lncRNAs may be related to allergy and iPSC-MSC immunomodulation. This study provides a key foundation to study the mechanisms underlying airway allergic inflammation and iPSC-MSC immunomodulation, which may be helpful in promoting iPSC-MSC medical applications.

## Additional file

Additional file 1: Supplementary data, figures and tables for The lncRNAs involved in mouse airway allergic inflammation following induced pluripotent stem cell-mesenchymal stem cell treatment. (DOCX 463 kb)

## Abbreviations

AHR: Airway hyper-reactivity
BM: Bone marrow
BM-MSC: Bone marrow-derived mesenchymal stem cell
BP: Biological process
CC: Cellular component
DC: Dendritic cell
GO: Gene ontology
H&E: Hematoxylin and eosin
IL: Interleukin
ILC2: Group 2 innate lymphoid cell
iPSC: Induced pluripotent stem cell
iPSC-MSC: Induced pluripotent stem cell-mesenchymal stem cell
lncRNA: Long noncoding RNA
Mch: Methacholine
MF: Molecular function
MSC: Mesenchymal stem cell
OVA: Ovalbumin
PAS: Periodic acid-Schiff
PBS: Phosphate-buffered saline
qRT-PCR: Quantitative real-time polymerase chain reaction
Th2: Type 2 helper T
Tm: Memory T
Treg: T regulatory
