# Supplementary material for: The lncRNAs involved in mouse airway allergic inflammation following induced pluripotent stem cell-mesenchymal stem cell treatment
Source: Stem Cell Res Ther. 2017 Jan 6;8:2. doi: 10.1186/s13287-016-0456-3 (PMC5216550; doi:10.1186/s13287-016-0456-3)
Supplement: Additional file 1: — Supplementary data, figures and tables for The lncRNAs involved in mouse airway allergic inflammation following induced pluripotent stem cell-mesenchymal stem cell treatment. (DOCX 463 kb) [file 13287_2016_456_MOESM1_ESM.docx]

**Additional file 1**

***Induction and culture of pluripotent stem cells-mesenchymal stem cells***

As in our previous study, *Effects of mesenchymal stem cells from human iPS cells on differentiation, maturation, and function of dendritic cells,* which is under reviewed in *Stem Cell Research & Therapy,* urine cell-derived-iPSCs (U-iPSCs) [1] donated by the Guangzhou Institute of Biomedicine and Health at the Chinese Academy of Science (Guangzhou, Guangdong, China) were used for the generation of MSCs. iPSCs were induced to the MSCs, as previously reported, with minor modifications [2, 3]. Briefly, the passaged iPSCs were kept until 60% confluency, and then were induced to generate MSCs for 2 weeks. The induced-cells were plated onto 0.1% gelatin-coated flasks and defined as passage 1 (P1) iPSC-MSCs. All cells were incubated at 37℃ and supplemented with 5% CO_2_. iPSC-MSCs exhibited a typical fibroblastic morphology similar to MSCs, they were positive for CD105, CD73, CD90, CD146, CD144 and CD44, and negative for CD34, CD14 and CD45. Tri-lineage differentiation experiments, including osteogenic, chondrogenic and adipogenic differentiation, were used to confirm the multipotency of iPSC-MSCs. For experimentation, the cells were used with passage numbers lower than 14, and cell growth densities were maintained at 70-80%.

***In vivo* *stu*dy**

The animal model was established according to our previous description with minor modifications [4, 5]. The mice were divided into three groups (n=15, 16, and 15 for PBS/PBS/PBS, OVA/OVA/PBS and OVA/OVA/iPSC-MSC). The mice were sensitized with an intraperitoneal (ip.) injection of 40 µg Ovalbumin (OVA) and 100 µl Inject Alum (Thermo Scientific, Rockford, USA) on day 1, day 7 and day 14. iPSC-MSCs were suspended in PBS were injected via the tail vein on day 20 before the first challenge. The suspended density of iPSC-MSCs was 5×10^6^ cells/ml, and a 0.2 ml cell suspension was injected per mouse. On days 21 to 24, the mice were challenged with aerosolized 5% OVA in a large, clear plastic container through an air-compressing nebulizer (403A, Yuyue, Danyang, Jiangsu, China) for 30 minutes. An equal amount of PBS was used for control in sensitization, challenge and treatment. The degree of airway responsiveness to methacholine (Mch) was measured 24 hours after the last challenge, as our previous study (n=6 for each group) [5]. The rest of the mice (n=9, 10, 9 for PBS/PBS/PBS, OVA/OVA/PBS and OVA/OVA/ iPSC-MSC) were sacrificed 6 hours after the last challenge. The middle lobes of the right lung (n=3 for each group) were used for RNA microarray extraction, and the other middle lobes of the right lung (n=6, 7, 6 for PBS/PBS/PBS, OVA/OVA/PBS and OVA/OVA/ iPSC-MSC) were used for qRT-PCR RNA extraction. The inferior lobes of the left lung (n=6 for each group) were stained with hematoxylin-eosin (HE) and periodic acid–Schiff (PAS, Baso Diagnostics Inc, Zhuhai, Guangdong, China) to assess the degree of airway inflammation. For a quantification of lung inflammation, the goblet cell counts and inflammatory infiltration scores in the lungs were performed as previously described [5].

***In vitro* study**

The mice (n=8) were sensitized by OVA on day 1 and day 7 and were sacrificed on day 14 to obtain the memory T (Tm) cells [6]. Briefly, the mouse spleens were sifted through a 40-μm mesh sieve to obtain the mononuclear cells for culture. The red blood cells were removed using a RBC Lysis Buffer lysis (eBioscince, San Diego, USA). The CD3+ T cells were positively selected from the mononuclear cells using the magnetic activated cell sorting (MACS) CD3 Microbeads (Miltenyi Biotec, Auburn, CA). To measure the cytokine secretion of memory T (Tm) cells, the Tm cells were divided into three groups (n=5 for each group): (1) Tm only group whose cells were cultured without an additional challenge; (2) Tm+OVA group whose cells were cultured and challenged with OVA (2 mg/ml); (3) Tm+OVA+iPSC-MSC whose cells were co-cultured with iPSC-MSCs at a rate of 10:1 and challenged with OVA (2 mg/ml) [7]. After 3 days, the suspended T cells were collected for a microarray analysis (n=3 mice for each group). Th2 cytokines including, IL-4 and IL-13 in culture supernatant (n=5 mice for each group), were measured with enzyme-linked immunosorbent assays (ELISA) following the manufacturer’s instructions (R&D Systems, Minneapolis, MN).

***RNA extraction and lncRNA quantification using qRT-PCR***

Total RNA was extracted with a Trizol reagent (Invitrogen, Paisley, UK), and cDNA was synthesized with the Takara PrimeScriptTM RT Master Mix Kit (Takara Bio, Otsu, Japan). lncRNAs were quantified using a quantitative real-time PCR (qRT-PCR) with the FastStart Universal SYBR Green Master (ROX) (Roche, Switzerland). The primer specificity of the lncRNA *mouselincRNA0307+* was not high enough, so only 8 lncRNAs were finally confirmed using qRT-PCR. The primers used in the qRT-PCR are shown in Table S1. RPS18 was detected as the internal control. After the qRT-PCR amplification, the melt curve was performed to confirm reaction specificity, and the fold change (FC) of each lncRNA was calculated via the 2-ΔΔCt method.

***References***

1. Xue Y, Cai X, Wang L, Liao B, Zhang H, Shan Y, Chen Q, Zhou T, Li X, Hou J, et al: **Generating a non-integrating human induced pluripotent stem cell bank from urine-derived cells.** *PLoS One* 2013, **8:**e70573.

2. Lian Q, Zhang Y, Zhang J, Zhang HK, Wu X, Zhang Y, Lam FF, Kang S, Xia JC, Lai WH, et al: **Functional mesenchymal stem cells derived from human induced pluripotent stem cells attenuate limb ischemia in mice.** *Circulation* 2010, **121:**1113-1123.

3. Li YP, Paczesny S, Lauret E, Poirault S, Bordigoni P, Mekhloufi F, Hequet O, Bertrand Y, Ou-Yang JP, Stoltz JF, et al: **Human mesenchymal stem cells license adult CD34(+) hemopoietic progenitor cells to differentiate into regulatory dendritic cells through activation of the notch pathway.** *Journal of Immunology* 2008, **180:**1598-1608.

4. Sun YQ, Deng MX, He J, Zeng QX, Wen W, Wong DS, Tse HF, Xu G, Lian Q, Shi J, Fu QL: **Human pluripotent stem cell-derived mesenchymal stem cells prevent allergic airway inflammation in mice.** *Stem Cells* 2012, **30:**2692-2699.

5. Yao Y, Zeng QX, Deng XQ, Tang GN, Guo JB, Sun YQ, Ru K, Rizzo AN, Shi JB, Fu QL: **Connexin 43 Upregulation in Mouse Lungs during Ovalbumin-Induced Asthma.** *PLoS One* 2015, **10:**e0144106.

6. Mozingo DW, Cairns BA, Farrell KJ: **Increased toll-like receptor 4 expression on T cells may be a mechanism for enhanced T cell response late after burn injury - Discussion.** *Journal of Trauma-Injury Infection and Critical Care* 2006, **61:**298-299.

7. Aggarwal S, Pittenger MF: **Human mesenchymal stem cells modulate allogeneic immune cell responses.** *Blood* 2005, **105:**1815-1822.

| **Table S1**  **The listed primers were used to validate the expression of the lncRNAs.** | | |
| --- | --- | --- |
| **Name** | **Forward primer** | **Reverse primer** |
| ENSMUST00000139014 | ACAGCTTGCACCCACTCTTT | GTGTGCCCTTCTGACCATCT |
| ENSMUST00000124434 | AGGACCTCTGTCTCCCCTTG | CTACTGTGTCCCCCATGGTC |
| ENSMUST00000050671 | AAAAGTGCCAGCTCCGACTC | CTGCTATCACCGCTGTTGCT |
| AK029213 | CTCCCATCTGTTTGCCTCAT | CTGGCTTTCTTGGGTACTGG |
| MM9LINCRNAEXON12105+ | GATTGAAGATTGATTGTTAAGCTG | TTGCAGTGCCTTCACTTGAG |
| ENSMUST00000162289 | GAATGGCAGTGTGGACCTCT | CGCTCTGTTATCCAGCTTCC |
| AK144717 | TGGACTGATGACTGACGACTG | GGCTGCTATCTGGAGTTGGA |
| AK089315 | TGAGTATCCCTGAGCCCTTG | TGATGACTACGCTGGCTTTG |
| RPS18 | ATAGCCTTCGCCATCACTGC | ATGGTGATCACTCGCTCCAC |

The primer information of the selected lncRNAs used in the qRT-PCR is provided; however, the primer specificity of one lncRNA, *mouselincRNA0307+,* was not high enough, so only 8 were shown.

| **Table S2**  **Twenty-three lncRNAs differentially expressed in the same trend, both in vivo and in vitro.** | | | | | |
| --- | --- | --- | --- | --- | --- |
| **Seqname** | **Source** | **Chrom** | **Strand** | **Txstart** | **Txend** |
| uc008efj.1 | UCSC_kg | chr18 | + | 21811063 | 21814606 |
| ENSMUST00000139014 | Ensembl | chr11 | - | 120091115 | 120092899 |
| mouselincRNA0307+ | lincRNA | chr12 | + | 29797876 | 29822578 |
| AK144501 | fantom3 | chr10 | - | 53018273 | 53018841 |
| ENSMUST00000124434 | Ensembl | chr6 | + | 17148121 | 17160152 |
| uc.428+ | UCR | chr18 | + | 21813453 | 21813692 |
| ENSMUST00000050671 | Ensembl | chr18 | + | 21810065 | 21814605 |
| AK029213 | fantom3 | chr19 | + | 12671541 | 12672799 |
| ENSMUST00000144146 | Ensembl | chr7 | - | 3813646 | 3820772 |
| uc009eex.1 | UCSC_kg | chr6 | - | 128901221 | 128925047 |
| AK082906 | fantom3 | chr10 | + | 51251182 | 51253848 |
| AK155801 | fantom3 | chr6 | + | 17067639 | 17068700 |
| BC040222 | NRED | chr19 | + | 3083614 | 3087407 |
| ENSMUST00000121574 | Ensembl | chr2 | - | 53555154 | 53555838 |
| MM9LINCRNAEXON12105+ | lincRNA | chr1 | + | 162967481 | 162967775 |
| ENSMUST00000121225 | Ensembl | chrX | + | 73602165 | 73602469 |
| AK135581 | fantom3 | chr9 | - | 59671126 | 59672409 |
| ENSMUST00000162289 | Ensembl | chr1 | + | 162965293 | 162968664 |
| uc007qai.1 | UCSC_kg | chr13 | - | 33654354 | 33660738 |
| AK035610 | fantom3 | chr13 | + | 16120652 | 16123187 |
| AK144717 | fantom3 | chr1 | + | 25072148 | 25075540 |
| uc007qnc.1 | UCSC_kg | chr13 | - | 52825336 | 52830974 |
| AK089315 | NRED | chr2 | - | 117940122 | 117951453 |

Detailed information, including seqname, source database, chromosome localization *etc.* for 23 selected differentially expressed lncRNAs.

**Additional figure**

**
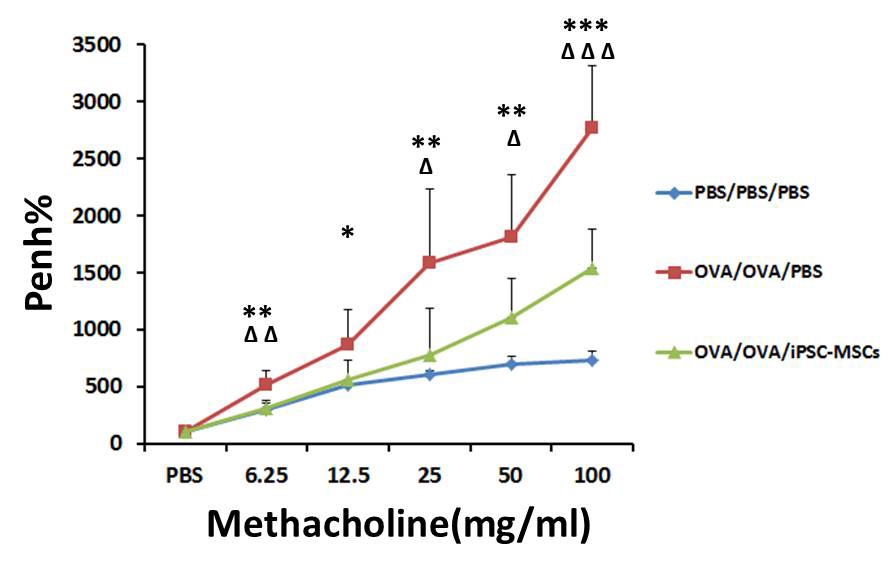
**

**Figure S1 IPSC-MSCs alleviated airway hyper-reactivity *in vivo*.** The OVA/OVA/PBS mice presented remarkably high airway responsiveness to increased methacholine (Mch) doses (6.25, 12.5, 25, 50 and 100 mg/ml) compared to PBS/PBS/PBS. iPSC-MSC treatment reduced the AHR of OVA/OVA/PBS group. * for comparison between OVA/OVA/PBS and PBS/PBS/PBS, Δ for comparison between OVA/OVA/iPSC-MSC and OVA/OVA/PBS. n=6, * and Δ for p value < 0.05, ** and ΔΔ for p value < 0.01, *** and ΔΔΔ for p value < 0.001.


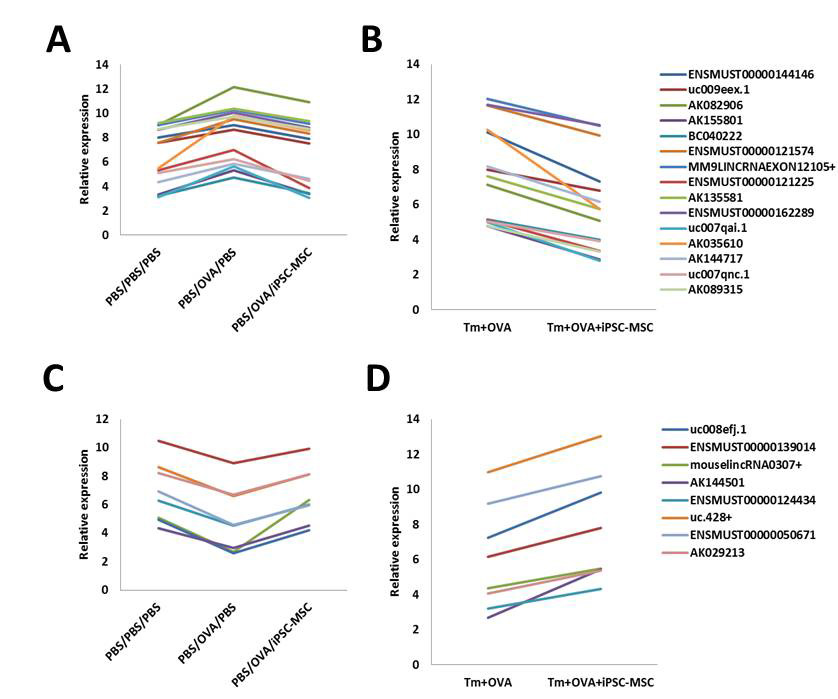


**Figure S2.** **The visual expression of selected 23 lncRNAs changed in two patterns as expected are shown.** (A, B): Fifteen lncRNAs were up-regulated in OVA/OVA/PBS and down-regulated in OVA/OVA/iPSC-MSC *in vivo* (A), and down-regulated in Tm+OVA+iPSC-MSC *in vitro* (B). (C, D): Eight lncRNAs were down-regulated in OVA/OVA/PBS and up-regulated in OVA/OVA/iPSC-MSC *in vivo* (C), and up-regulated in Tm+OVA+iPSC-MSC *in vitro* (D). (n=3)

**
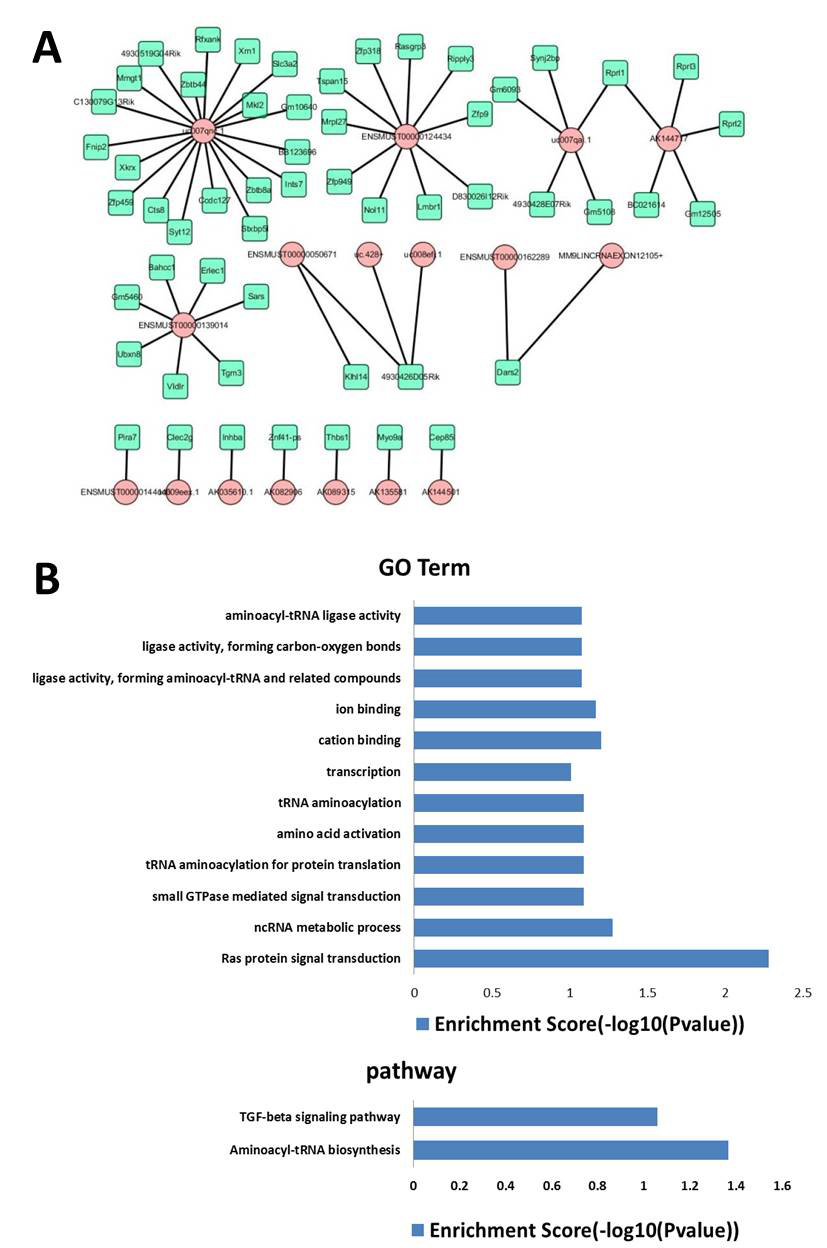
**

**Figure S3. Fifty-eight genes were predicted as targets of 23 lncRNAs that were selected and analysed for functional enrichment.** (A): The network showed the connection of 23 lncRNAs and their58 predicted targets. The pink nodes denote lncRNAs, and the green nodes denote mRNAs. (B): The GO and pathway analysis of the 58 genes predicted are shown. All GO ontologies and pathways involved are shown.
